# Intrathecally Administered Apelin-13 Alleviated Complete Freund’s Adjuvant-Induced Inflammatory Pain in Mice

**DOI:** 10.3389/fphar.2020.01335

**Published:** 2020-08-28

**Authors:** Shuangyu Lv, Xiaomei Zhang, Yuchen Zhou, Yu Feng, Yanjie Yang, Xinchun Wang

**Affiliations:** ^1^Institute of Molecular Medicine, School of Basic Medical Sciences, Henan University, Kaifeng, China; ^2^Key Laboratory of Clinical Resources Translation, The First Affiliated Hospital of Henan University, Kaifeng, China

**Keywords:** apelin, inflammatory pain, NMDA receptor, spinal cord, Fos

## Abstract

Apelin is the endogenous ligand for APJ, a G-protein-coupled receptor. Apelin gene and protein are widely distributed in the central nervous system and peripheral tissues. The role of apelin in chronic inflammatory pain is still unclear. In the present study, a mouse model of complete Freund’s adjuvant (CFA)-induced inflammatory pain was utilized, and the paw withdrawal latency/threshold in response to thermal stimulation and Von Frey ﬁlament stimulation were recorded after intrathecal (i.t.) injection of apelin-13 (0.1, 1, and 10 nmol/mouse). The mRNA and protein expression, concentration of glutamic acid (Glu), and number of c-Fos immunol staining in lumbar spinal cord (L4/5) were determined. The results demonstrated that *Apln* gene expression in the lumbar spinal cord was down-regulated in the CFA pain model. Apelin-13 (10 nmol/mouse, i.t.) alleviated CFA-induced inflammatory pain, and it exhibited a more potent antinociceptive effect than apelin-36 and (pyr)apelin-13. The antinociception of apelin-13 could be blocked by APJ antagonist apelin-13(F13A). I.T. apelin-13 attenuated the increased levels of *Aplnr*, *Grin2b, Camk2d*, and *c-Fos* genes expression, Glu concentration, and NMDA receptor 2B (GluN2B) protein expression caused by CFA. Apelin-13 significantly reduced the number of Fos-positive cells in laminae III and IV/V of the dorsal horn. This study indicated that i.t. apelin-13 exerted an analgesic effect against inflammatory pain, which was mediated by activation of APJ, and inhibition of Glu/GluN2B function and neural activity of the spinal dorsal horn.

## Introduction

Apelin, an endogenous peptide, was identified as the natural ligand of the orphan receptor APJ (Tatemoto et al., 1998). The APJ (putative receptor protein related to the type 1 angiotensin receptor) is a G-protein-coupled receptor (GPCR) and was cloned from a human genomic library cDNA in 1993 (O’Dowd et al., 1993). Apelin was initially isolated from bovine stomach extracts and was given the name apelin (Tatemoto et al., 1998). The human *Apln* gene is located on chromosome Xq25-26 and has 3 exons and 2 introns (Lee et al., 2000). Apelin is generated from a 77-amino-acid precursor, named preproapelin, which can be hydrolyzed by endopeptidases into several active biological fragments, including apelin-36 (apelin42–77), apelin-17 (apelin61–77), apelin-16 (apelin62–77), apelin-13 (apelin65–77), and apelin-12 (apelin66–77) and pyroglutaminated apelin-13 [(pyr)apelin-13] (Kawamata et al., 2001). Preproapelin contains N and C termini with potential proteolytic cleavage sites, and the sequence of 23 amino acids between tryptophan 55 and phenylalanine 77 is fully conserved among different species (Medhurst et al., 2003). Among these, apelin-13 is the most potent activator for APJ expressed in cell lines, and apelin-13 and apelin-36 are the most widely studied (Tatemoto et al., 1998; Habata et al., 1999; Kawamata et al., 2001).

The apelin/APJ system is involved in a broad range of physiological functions and pathological processes, including cardiovascular function (Wysocka et al., 2018; Esmaeili et al., 2019), cardiac contractility (Zhong et al., 2017; Esmaeili et al., 2019), angiogenesis (Wu et al., 2017; Cheng et al., 2019), energy metabolism (Bertrand et al., 2015; Castan-Laurell et al., 2019), liver diseases (Principe et al., 2008; Lv et al., 2017), ischemia/reperfusion injury (Yang et al., 2015; Chen et al., 2016) and cancer (Yang et al., 2016a; Masoumi et al., 2020). Recent studies were focused on the role of apelin in psychosis and neuropathy (Lv et al., 2020). Xiao et al. (2018) demonstrated that intrahippocampal infusion of apelin-13 (1-4 μg/rat) exhibited an anti-depressive effect in forced swim test. Chronic intracerebroventricular (i.c.v.) administration of apelin-13 (2 μg/d) alleviated chronic stress-induced depression-like phenotypes by ameliorating hypothalamic–pituitary–adrenal (HPA) axis and hippocampal glucocorticoid receptor dysfunction in rats (Dai et al., 2018). Peripheral injection of apelin-13 induced anxiolytic activity in a mouse model of chronic normobaric hypoxia by suppressing the nuclear factor κB (NF-κB) pathway, and silent mating type information regulation 2 homolog 1 (SIRT1) was involved in this process (Fan et al., 2017; Fan et al., 2018). In addition, the apelin/APJ system had a protective effect on memory impairment (Li et al., 2016; Haghparast et al., 2018), ischemic stroke (Yang et al., 2016b; Wu et al., 2018), and brain damage (Bao et al., 2015; Chu et al., 2016). However, the role of apelin in inflammatory pain is still unclear.

Apelin/APJ gene and protein are abundantly distributed in the central nervous system (CNS) and peripheral tissues in humans and rodents. In the periphery, apelin and APJ were expressed in lungs, heart, kidneys, stomach and intestine (Hosoya et al., 2000; Lee et al., 2000; O’Carroll et al., 2000). In the CNS, they were found in the spinal cord, dorsal raphe nucleus, amygdala and hypothalamus (Hosoya et al., 2000; O’Carroll et al., 2000; Reaux et al., 2001), which are the major regions closely related to pain intensity. The distribution pattern suggests that the apelin/APJ system plays a potential role in regulating pain. Our previous studies showed that i.c.v. administration of apelin-13 (0.5, 1 and 3 μg/mouse) induced an antinociceptive effect in a visceral pain model using acetic acid-induced writhing test (Lv et al., 2012), and apelin-13 infused intrathecally (i.t.) induced an antinociceptive effect in an acute pain model using tail immersion test (Lv et al., 2013). Chronic inflammatory pain is a common clinical disease characterized by persistent spontaneous pain and hyperalgesia. It is necessary to explore the regulatory effect of apelin/APJ on chronic inflammatory pain.

Infusion of complete Freund’s adjuvant (CFA) into rodents’ hind paw is considered to be a valid model to investigate the mechanism of chronic inflammatory pain, as well as to screen for anti-inflammatory hyperalgesic drugs (Beyer et al., 1997; Sun et al., 2012). The present study was designed to study the effect and mechanism of i.t. treatment with apelin on chronic inflammatory pain using a model induced by intraplantar injection of CFA, real-time quantitative polymerase chain reaction (qPCR), western blotting, high performance liquid chromatography (HPLC), and immunohistochemistry.

## Materials and Methods

### Animals

Male Kunming mice (aged 6–8 weeks) were supplied by the Animal Center of Henan Province (Zhengzhou, China). The animals were housed under controlled conditions with a 12:12 h light/dark cycle with food and water available *ad libitum*. Behavioral testing was performed from 09:00 to 15:00 h in a quiet room after animals had been acclimatized to the environment for at least 30 min. The animal experimental protocol was approved by the Committee of Medical Ethics and Welfare for Experimental Animals, Henan University School of Medicine. All efforts were made to minimize animal suffering and the number of the animals used in the following experiments.

### Chemicals and I.T. Injection

Apelin-13, (pyr)apelin-13, apelin-36, and apelin-13(F13A) were purchased from GL Biochem (Shanghai) Ltd. (Shanghai, China). The peptides were dissolved and diluted in physiological saline before treatment. The CFA was supplied by Sigma–Aldrich (St. Louis, MO, USA).

To evaluate the roles of spinal apelin in regulating inflammatory pain, peptide or antagonist was administered by i.t. delivery, as described by Hylden and Wilcox (Hylden and Wilcox, 1980). A stainless needle connected to a Hamilton microsyringe (25 μl) was inserted into the L5/6 intervertebral space in conscious mice. The reflexive lateral flick of the tail or formation of an S shape by the tail indicated successful treatment. The drug solution or vehicle was delivered in a total volume of 5 μL for 10 s. The correctness of the i.t. injection site was conﬁrmed by injecting an appropriate volume of methylene blue solution (1.0%).

### Behavioral Analysis

To induce a model of inﬂammatory pain, CFA was injected into the plantar surfaces of hind paws of the mice in a volume of 10 μL. The vehicle control group was injected with an equivalent volume of saline. The pain sensitivity was determined blindly as previous report (Wang et al., 2015). Heat hyperalgesia was assessed by evaluating the paw withdrawal latency (PWL) in response to a radiant heat source. The mice were placed in plastic boxes and allowed to be acclimatized for 30 min before the test. PWL was tested using the Thermal Stimulator System (PL-200; TaiMeng Technology Corporation, Chengdu, China). The animals were placed on a clear glass plate, and a radiant heat stimulus using a projector lamp bulb (8 V, 50 W) was focused on the plantar surface of each hind paw, with a cutoff of 10 s. The duration between the start of heat application and paw withdrawal was calculated as the PWL.

Mechanical allodynia was assessed by analyzing 50% paw withdrawal threshold (PWT) in response to Von Frey ﬁlament (Stoelting Co., Wood Dale, IL, USA) stimulation. Mice were put in boxes on an elevated metal mesh floor and allowed to habituate for 30 min before the test. The Von Frey ﬁlaments were applied perpendicularly to the central region of the plantar surface of one hind paw until the ﬁlaments were bent. The 50% PWT was measured using Dixon’s up–down method.

### Real-Time qPCR

Total RNA was extracted from lumbar spinal cord (L4/5) using TRIzol reagent. RNA was quantified by NanoDrop 2000 UV-Vis Spectrophotometer (Thermo Scientific, Wilmington, DE, USA). Total RNA (0.5 μg) from each mouse sample was reverse transcribed to synthesize cDNA using the High Capacity cDNA Reverse Transcription kit (Applied Biosystems, Foster City, CA, USA). The mRNAs level of apelin (*Apln*), apelin receptor (*Aplnr*), NMADR2A (*Grin2a*), NMDAR2B (*Grin2b*), CamK II (*Camk2d*), FBJ osteosarcoma oncogene (*Fos*), cAMP-response element binding protein 1 (*Creb1*), down-regulator of transcription 1 (*Dr1*), and Early growth response 1 (*Egr1*) were measured by real-time qPCR. Real-time PCR was carried out using the 7500HT Thermal Cycler and SYBR Green Master Mix (Applied Biosystems). Primers used were supplied by Wuhan Protein Interaction Bio Co. Ltd. (Wuhan, China), and they were designed as described previously (Kasai et al., 2010; Ze et al., 2014). Dissociation curve analysis was completed after each real-time qPCR. Ct values of mRNA expression of targeted genes were normalized to *36B4*. The relative fold change of target genes was analyzed by the equation 2^−ΔΔCt^. Sequences of the primers used for real-time qPCR was listed in **Table 1**.

**Table 1:** Primer sequence used for RT-qPCR.

Primers name	Primer sequence	Size (bp)
*Apln*-F	5’- GTTGCAGCATGAATCTGAGG-3’	247
*Apln*-R	5’- CTGCTTTAGAAAGGCATGGG-3’	
*Aplnr*-F	5’-CCACCTGGTGAAGACTCTCTACA-3’	110
*Aplnr*-R	5’- TGACATAACTGATGCAGGTGC-3’	
*Grin2a*-F	5’-ATGAACCGCACTGACCCTAAG-3’	246
*Grin2a*-R	5’-GGCTTGCTGCTGGATGGA-3’	
*Grin2b*-F	5’-AATGTGGATTGGGAGGATAGG-3’	255
*Grin2b*-R	5’-ATTAGTCGGGCTTTGAGGATACT-3’	
*Camk2d*-F	5’- AGAAGTTCAAGGCGACCAGCA -3’	150
*Camk2d*-R	5’- GGGTATCCCACCAGCAAGATGTAG -3’	
*c-Fos*-F	5’-GGTGAAGACCGTGTCAGGAGGCAG-3’	117
*c-Fos*-R	5’-GCCATCTTATTCCGTTCCCTTCGG-3’	
*Creb1*-F	5’-TACGGATGGGGTACAGGGC -3’	197
*Creb1*-R	5’-CAATGGTGCTCGTGGGTG -3’	
*Dr1*-F	5’-CTGGGAGTGGTGTCCCTAGA-3’	479
*Dr1*-R	5’-GCCCAAACTTTCCAGTGCTTG-3’	
*Egr1*-F	5’-GAGCACCTGACCACAGAGTC-3’	172
*Egr1*-R	5’-AAAGGGGTTCAGGCCACAAA-3’	
*36B4*-F	5’-CGACCTGGAAGTCCAACTAC-3’	109
*36B4*-R	5’-ATCTGCTGCATCTGCTTG-3’	

F, forward; R, reverse.

### Western Blotting

The mouse L4/5 spinal segments were dissected out and homogenized with RIPA lysis buffer in the presence of protease inhibitor (Beyotime, Shanghai, China). After centrifugation of the lysates (14,000 *g*, 10 min at 4°C), the protein concentration was determined by Bicinchoninic Acid Protein Assay Kit (Beyotime). Protein samples were loaded into each well, separated by sodium dodecyl sulfate-polyacrylamide gel electrophoresis for 40 min at 120 V, and then blotted onto polyvinylidene fluoride membranes for 70 min at 120 V. The blots were blocked with 5% milk at room temperature for 1 h, and then membranes were incubated with the rabbit antibodies against N-methyl-D-aspartate receptor (NMDAR)2A and NMDAR2B (1:1000, Abcam, Cambridge, MA, USA) at 4°C overnight. Thereafter, they were incubated with horseradish-peroxidase-conjugated secondary antibody (Proteintech, Wuhan, China) for 1 h. The relative intensities of target proteins were normalized using β-actin (1:1000, Beyotime) as an internal control. The membranes were incubated with enhanced chemiluminescent substrates (Thermo Scientific, Wilmington, DE, USA) and detected using automatic multifunction chemiluminescent detection system (Tanon, Shanghai, China). The protein levels were assessed by densitometry using Image-J Software.

### HPLC

The lumbar spinal cord (L4/5) was isolated and homogenized with cold PBS. The protein was precipitated by methanol, and the supernatants were obtained after centrifugation (10,000 *g*, 15 min). The content of Glu was detected by Qiangdao Sci-tech Innovation Testing Limited Company (Qingdao, China). The samples were analyzed using LC-10A HPLC system (Shimadzu, Japan), with L-homoserine as an internal standard. Reversed phase column (ODS-C18, 250×4 mm) was used at 38°C. The excitation wavelength and emission wavelength of the fluorescent detector were set at 340 nm and 450 nm, respectively. The mobile phase was methanol: water (50: 50) at a flow rate of 1.0 mL/min. The samples were mixed with L-homoserine and o-phthalaldehyde for 2 min, and the injection volume was 10 μL.

### Immunohistochemistry

After the behavior test, mice were immediately anesthetized with pentobarbital sodium (100 mg/kg, intraperitoneally). Animals were perfused transcardially and fixed in 4% paraformaldehyde in 0.1 M phosphate buﬀer (pH 7.4). Each spinal cord was instantly removed, fixed with the above fixative overnight, and then embedded in paraffin. Transverse sections (5 μm thick) of the L4/5 spinal cord were cut using a vibratome (Leica Biosystems, Nussloch, Germany), and stained as previously described (Bao et al., 2017). Serial sections were blocked in 10% normal goat serum at room temperature for 1 h. A rabbit anti-c-Fos antibody (1:100, Abcam Inc., Burlingame, CA, USA) was applied and sections were incubated overnight at room temperature. Following incubation with a biotinylated secondary antibody (Proteintech, Wuhan, China) for 2 h, all sections were processed with the avidin–biotin–peroxidase complex (Corning Inc., Corning, NY, USA) for 30 min. The results of the immunostaining were revealed by 3,3-diaminobenzidine kit (ZSGB-bio, Beijing, China). To calculate in detail, spinal dorsal horn was divided into three areas, including laminae I/II (superﬁcial dorsal horn), laminae III/IV (nucleus proprius), and laminae V/VI (neck of the dorsal horn). These areas were selected because they are crucial for nociceptive transmission in the dorsal horn (Besson and Chaouch, 1987). The left (ipsilateral) side of each section was used for data analysis, and the quantity of c-Fos-like immunoreactive (FLI) cells was assessed by Image-J.

### Data Analysis

All values are expressed as mean ± SEM. Data were analyzed using one-way analysis of variance followed by Dunnett’s test for *post hoc* comparisons. A two-tailed un-paired Student’s *t* test was performed to evaluate the difference between the two groups. The level of signiﬁcance was set at *p* < 0.05.

## Results

### Relative *Apln* mRNA Level Was Decreased in CFA-Treated Mice

**Figure 1:**
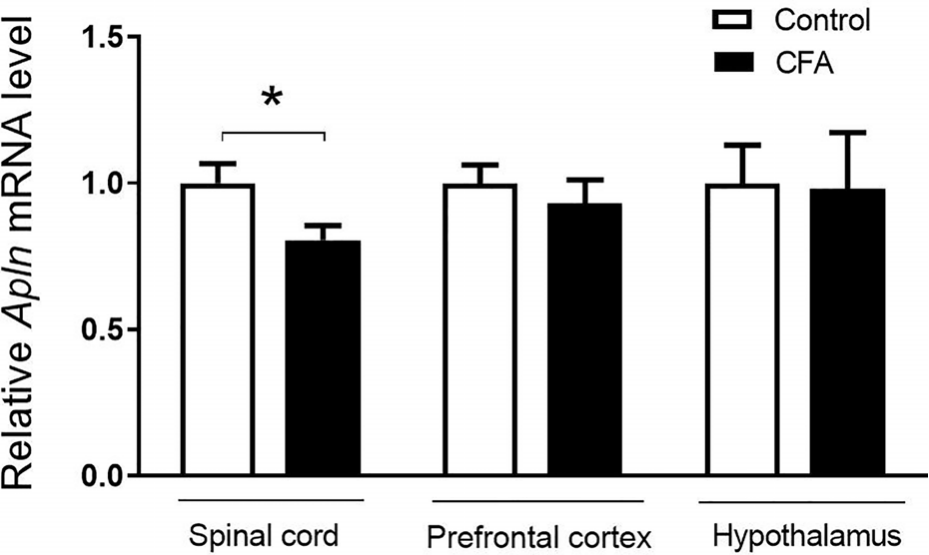
*Apln* gene expression in the CFA-induced inﬂammatory pain in mice. mRNA levels in L4/5 spinal cord, prefrontal cortex and hypothalamus were detected by real-time qPCR, normalized with the housekeeping gene *36B4*. Data are expressed as means ± SEM. n = 8 or 9 per group. *p < 0.05, compared with vehicle control.

To examine the possible changes of *Apln* gene between CFA-induced inflammatory pain model and vehicle control, the tissues, including L4/5 spinal cord, prefrontal cortex, hippocampus, and hypothalamus, were collected and mRNAs were determined. As shown in **Figure 1**, *Apln* mRNA was significantly decreased in L4/5 spinal cord of the CFA-induced inflammatory hyperalgesia mouse model, compared with the vehicle control (*p* < 0.05). However, *Apln* mRNA in prefrontal cortex (*p* = 0.505) or hypothalamus (*p* = 0.936) was not changed.

### I.T. Application of Apelin Alleviated Inﬂammatory Pain

**Figure 2:**
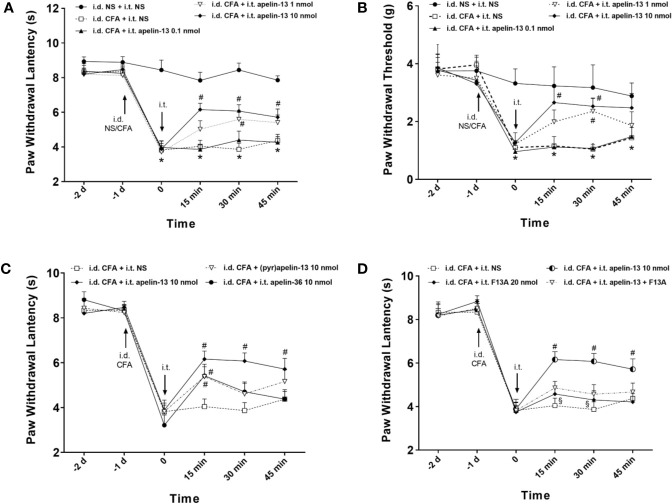
I.T. injection of apelin alleviated the inﬂammatory pain induced by intradermal injection of CFA in mice. Normal saline (NS) was used as a control. The upward and downward arrows indicate the time points when i.d. and i.t. injection were administered. **(A, B)** Effect of apelin-13 (0.1, 1 and 10 nmol/mouse, i.t.) on PWL in response to thermal stimulation and PWT in response to Von Frey ﬁlament stimulation. **(C)** The effect of i.t. application of 10 nmol apelin-13, (pyr)apelin-13 and apelin-36 on PWL. **(D)** Effect of APJ receptor antagonist apelin-13(F13A) (20 nmol/mouse) on antinociception of (pyr)apelin-13 (10 nmol/mouse). Data are expressed as means ± SEM. n = 6–10 per group. *p < 0.05, compared with vehicle control (i.d. NS + i.t. NS); ^#^*p* < 0.05, compared with CFA group (i.d. CFA + i.t. NS); ^§^*p* < 0.05, compared with apelin-13-treated group (i.d. CFA + i.t. apelin-13). F13A, apelin-13(F13A).

To explore the effect of apelin-13 on inﬂammatory pain, CFA was injected subcutaneously into the plantar surface of the left hind paws of mice, and the responses to painful stimuli were detected. As expected, CFA injection induced hypersensitivity, which presented as reduced PWL in response to thermal stimuli (**Figure 2A**) and reduced PWT of mice in response to mechanical stimuli (von Frey assay) (**Figure 2B**). Apelin or saline were i.t. injected at 24 h after CFA treatment, and dose of apelin-13 was selected according to the previous report (Lv et al., 2013). Apelin-13 at the dose of 10 nmol/mouse significantly elevated the PWL of CFA-injected mice at 15, 30 and 45 min (each *p* < 0.05), and increased PWT values at 15 and 30 min (each *p* < 0.05). In addition, 1 nmol apelin-13 increased the PWL and PWT values of CFA-injected mice at 30 min (each *p* < 0.05). These results indicate that i.t. apelin-13 ameliorated CFA-induced hypersensitivity in response to mechanical and thermal stimuli in mice.

To compare the analgesic effect of different fragments of apelin, apelin-36, apelin-13 and (pyr)apelin-13 were i.t. administered at 10 nmol/mouse, and the nociceptive response was evaluated. Our result demonstrated that apelin-36, apelin-13 and (pyr)apelin-13 produced an obvious increase in PWL values of CFA-injected mice at 15 min (each *p* < 0.05), indicating an antinociceptive effect of these three forms of apelin (**Figure 2C**). Among them, apelin-13 exhibited the most potent analgesic effect in the CFA-induced inflammatory pain mouse model.

### APJ Was Involved in Antinociceptive Effect of I.T. Apelin-13

To verify whether APJ was involved in the analgesic effect of apelin-13, the specific APJ antagonist apelin-13(F13A) was selected. Apelin-13(F13A) (i.t., 20 nmol/mouse) had no influence on the PWL values of CFA-injected mice (**Figure 2D**). However, it significantly blocked the increased PWL values induced by apelin-13 (10 nmol/mouse) in CFA-treated mice at 15 min and 30 min (each *p* < 0.05, compared with apelin-13 treated group). These results indicated that the inhibitory effect of apelin-13 on CFA-induced hyperalgesia was mediated by APJ.

**Figure 3:**
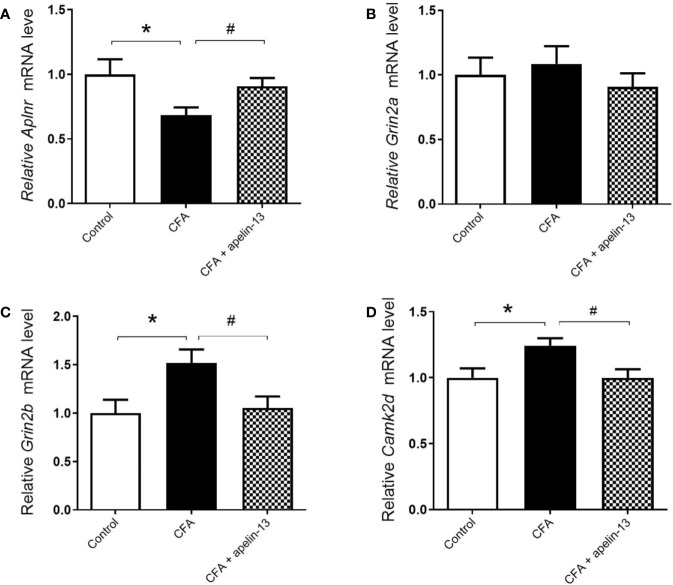
Effect of i.t. apelin-13 on gene expression in the L4/5 spinal cord of mice. mRNA expression levels of *Aplnr* **(A)**, *Grin2a* **(B)**, *Grin2b* **(C)** and *CamK2d* **(D)** were normalized with the housekeeping gene *36B4* mRNA expression using real-time qPCR. Data are expressed as means ± SEM. n = 6–9 per group. *p < 0.05, compared with vehicle control (i.d. NS + i.t. NS); ^#^*p* < 0.05, compared with CFA group (i.d. CFA + i.t. NS).

To further determine whether apelin-13 influence *Aplnr* gene expression, the relative *Aplnr* mRNA level in lumbar (L4/5) spinal cord was detected. As shown in **Figure 3A**, the *Aplnr* mRNA level significantly reduced in CFA treated mice (*p* < 0.05, compared with control), which was significantly reversed by i.t. apelin-13 (compared with apelin-13 treated group), suggesting the involvement of APJ in the antinociception of apelin-13.

### Apelin-13 Reduced *Grin2b* and *Camk2d* mRNAs in Mice With CFA-Induced Inflammatory Pain Model

To explore whether the key molecules, NMADR2A (NR2A), NMDAR2B (NR2B) and CamK II were involved in pain transmission for apelin-13, the relative mRNAs level of *Grin2a*, *Grin2b*, and *Camk2d* in the mouse lumbar (L4/5) spinal cord were determined. The results demonstrated that CFA or apelin-13 did not affect *Grin2a* gene expression (*p* = 0.662, compared with control; *p* = 0.331, compared with CFA treated group, **Figure 3B**). However, CFA caused an increase of *Grin2b* and *Camk2d* gene expression, compared with saline-treated group (each *p* < 0.05) (**Figures 3C, D**). The increased gene expression was significantly reduced after i.t. apelin-13 (each *p* < 0.05, compared with CFA treated group, **Figures 3C, D**).

### Apelin-13 Mitigated the Elevated NR2B Expression and Glu Concentration Induced by CFA

**Figure 4:**
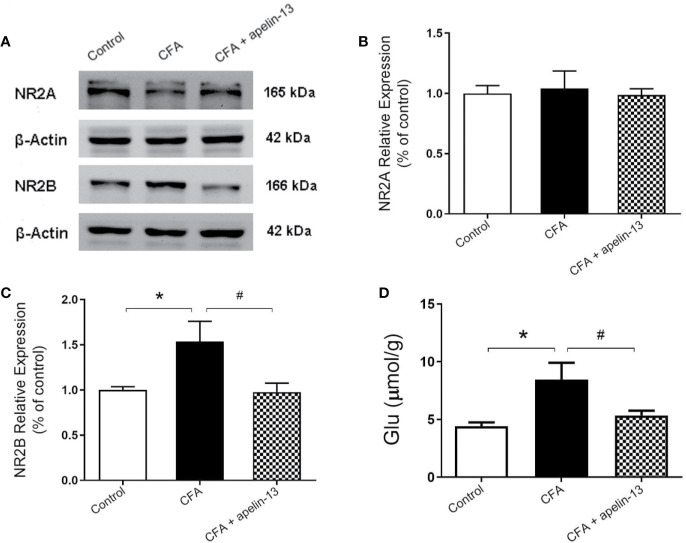
Effect of i.t. apelin-13 on NR2A and NR2B expression, and Glu concentration in mice. **(A)** Expression of NR2A and NR2B in the mouse L4/5 spinal cord determined by western blotting. β-Actin was used as a loading control. **(B, C)** Quantitative analysis of normalized optical density (NR2A/β-actin, NR2B/β-actin) in the three groups. **(D)** Concentration of Glu in the mouse L4/5 spinal cord detected by HPLC. Data are expressed as means ± SEM. n = 4–6 per group. *p < 0.05, compared with vehicle control (i.d. NS + i.t. NS); ^#^*p* < 0.05, compared with CFA group (i.d. CFA + i.t. NS).

To confirm the involvement of NR2A/B in the antinociception of apelin-13, the relative protein expression was detected using western blotting. As shown in **Figures 4A–C**, CFA induced an increase in GluN2B expression in the mouse lumbar spinal cord (*p* < 0.05, compared with control), but not GluN2A (*p* = 0.80, compared with control). The elevated GluN2B expression was significantly reduced by i.t. aplein-13 (*p* < 0.05, compared with CFA treated group). In addition, to measure the change of Glu, a neuroexcitatory neurotransmitter, in the lumbar spinal cord, the free Glu was detected by HPLC. The concentration of Glu was significantly induced by CFA compared with the control group (*p* < 0.05), which significantly decreased after apelin-13 treatment, compared with the CFA group (*p* < 0.05, **Figure 4D**).

### Apelin-13 Reduced *Fos* Gene Expression and the Number of Fos-Positive Cells in Laminae III and IV/V of the Dorsal Horn

**Figure 5:**
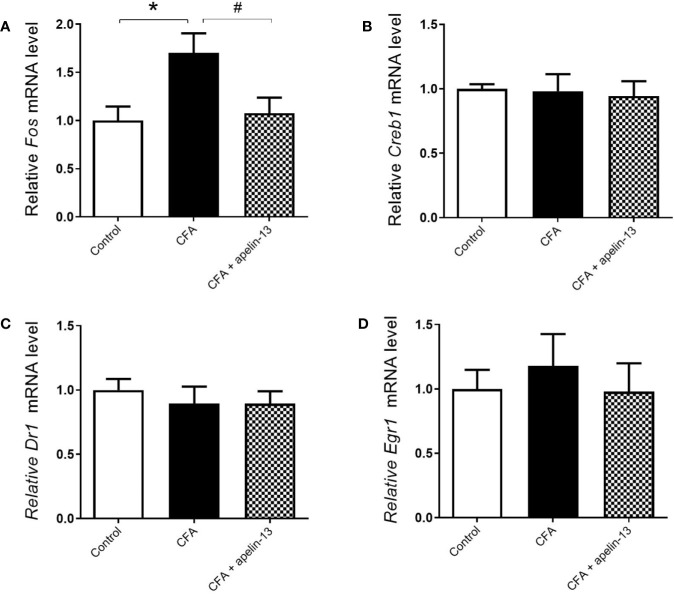
Effect i.t. apelin-13 on the *c-Fos*, *Creb1*, *Dr1* and *Egr1* gene expression in mouse L4/5 spinal cord. mRNA expression levels of *Fos* **(A)**, *Creb1* **(B)**, *Dr1* **(C)** and *Egr1* **(D)** were determined by real-time qPCR and normalized with the housekeeping gene *36B4* mRNA expression. Data are expressed as means ± SEM. *p < 0.05, compared with vehicle control (i.d. NS + i.t. NS); ^#^*p* < 0.05, compared with CFA group (i.d. CFA + i.t. NS). n = 8 or 9 per group.

To verify whether the transcription factors were involved in the antinociception of apelin-13, the mRNAs level of *c-Fos*, *Creb1*, *Dr1*, and *Egr1* were measured. Compared with the saline-treated control group, CFA obviously up-regulated *c-Fos* gene expression in the mouse lumbar spinal cord (*p* < 0.05, **Figure 5A**). However, it had no influence on *Creb1* (*p* = 0.89, **Figure 5B**), *Dr1* (*p* = 0.51, **Figure 5C**) and *Egr1* (*p* = 0.54, **Figure 5D**) gene expression. The increased *c-Fos* mRNA was significantly down-regulated by apelin-13 (*p* < 0.05).

**Figure 6:**
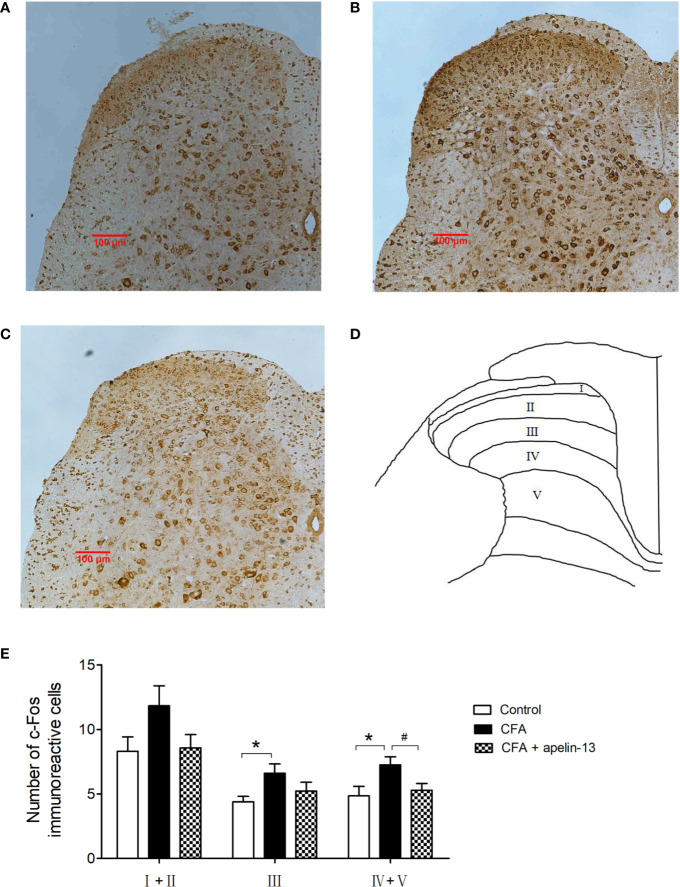
Effect of apelin-13 on Fos protein expression in L4/5 spinal cord in mice. Representative sections of the lumbar spinal cord showing Fos-positive cells in the vehicle control (i.d. NS + i.t. NS, **A**), CFA group (i.d. CFA + i.t. NS, **B**) and apelin-13-treated group (i.d. CFA + i.t. NS, **C**). **(D)** Objective to measure and evaluate the distribution of Fos-positive cells of the lumbar spinal cord in mice. **(E)** Quantification of the number of Fos-positive cells in the spinal cord L4/5 segments from mice. Data are expressed as means ± SEM. *p < 0.05, compared with vehicle control (i.d. NS + i.t. NS); ^#^*p* < 0.05, compared with CFA group (i.d. NS + i.t. CFA). n = 6 per group. Scale bars = 100 μm.

To study the role of apelin in regulating chronic inflammatory pain, CFA-induced Fos expression was used as a functional marker to identify the activation of spinal neurons. Intradermal (i.d.) injection of CFA into the hind paw of the mice stimulated Fos expression within the L4/5 segments of the ipsilateral side of the spinal cord (**Figures 6A–E**). Compared with the control group, the number of FLI neurons in the CFA-treated group reached statistical significance in laminae III (*p* < 0.05) and laminae IV/V (*p* < 0.05), but not in laminae I/II (*p* = 0.10, **Figure 6E**). The elevated number of Fos-labeled neurons induced by CFA was reduced by i.t. apelin-13 in laminae IV/V (*p* < 0.05), but not in laminae III (*p* = 0.19).

## Discussion

Our present study indicated that *Apln* mRNA was down-regulated in the lumbar spinal cord of mice with inflammatory pain induced by CFA, compared with the vehicle control group. Apelin-13 (1 and 10 nmol/mouse, i.t.) alleviated CFA-induced hypersensitivity to both mechanical stimuli and thermal stimuli. Apelin-13 exerted more potent analgesic activity than apelin-36 and (pyr)apelin-13 in the CFA-induced inflammatory pain model. Wang et al. found that electroacupuncture stimulation alleviated CFA-induced inflammatory pain by restoring apelin and APJ mRNA and protein expression (Wang et al., 2016), suggesting that the apelin/APJ system has a close relationship with inflammatory pain. The present result confirmed that apelin induced an inhibitory effect on chronic inflammatory pain induced by CFA at the spinal level. Our previous studies showed that i.c.v. or i.t. apelin-13 inhibited acute pain in acid-induced writhing and tail immersion tests (Lv et al., 2012; Lv et al., 2013). In addition, it was reported that chronic injection of (pyr)apelin-13 (i.t., 1 and 5 μg/rat) ameliorated neuropathic pain after spinal cord injury (Xiong et al., 2017), and APJ antagonist ML221 mitigated neuropathic pain induced by chronic constriction injury (Hajimashhadi et al., 2017). It was reported that chronic apelin (3 μg/rat, i.t.) produced thermal antinociception and down-regulated spinal APJ. However, apelin could induce tolerance to its antinociceptive effect (Abbasloo et al., 2016). These results demonstrate that apelin induces consistent analgesic effects in different types of pain models.

The apelin receptor, APJ, shares 40–50% sequence homology with angiotensin II type 1 receptor, but it does not bind to angiotensin II (O’Dowd et al., 1993). Given the wide range of tissue distribution and physiological functions of APJ, it is considered as an interesting target. The specific APJ receptor antagonist was designed by mutation of the carboxyl-terminal phenylalanine, named apelin-13(F13A), and it blocked the hypotensive effects of apelin-13 (Lee et al., 2005). Our results indicated that apelin-13(F13A) significantly antagonized the inhibitory effect of apelin-13 on hyperalgesia response induced by CFA, whereas, apelin-13(F13A) alone did not influence CFA-induced hyperalgesia. Additionally, the down-regulated *Aplnr* mRNA in the CFA-treated group was restored by apelin-13 infusion. These results demonstrated that the antinociception of apelin-13 was mediated by APJ, which was supported by the anatomical site of APJ, such as the spinal cord (Hosoya et al., 2000; O’Carroll et al., 2000).

NMDARs, as glutamate-gated ion channels, play a key role in regulating synaptic plasticity. NMDARs consisted of three homologous subunits, including GluN1/NR1, GluN2/NR2 (GluN2A–GluN2D), and GluN3/NR3 (GluN3A–GluN3B). The major NMDAR subtypes in spinal dorsal horn are GluN1 (NR1A), GluN2A (NR2A), and GluN2B (NR2B) subunits. Our result showed that the gene and protein levels of GluN2B in lumbar spinal cord were significantly increased in the mouse model of CFA-induced inflammatory pain, compared with the control group. This is supported by the established theory that peripheral inﬂammation causes speciﬁc accumulation of NR2B receptors at spinal cord synapses (Tan et al., 2005; Yang et al., 2009; Zhuo, 2009). In the present study, i.t. apelin-13 attenuated the elevated NR2B gene and protein expression in the lumbar spinal cord of mice with CFA-induced chronic inflammatory pain, indicating that NR2B was involved in the antinociception of apelin-13. It has been proved that glutamate is a crucial transmitter of excitatory pathways to the spinal cord (Gougis et al., 2002). HPLC analysis showed that apelin-13 diminished the up-regulated glutamate level induced by CFA in mouse lumbar spinal cord. We suppose that the antinociception of apelin-13 may be caused by inhibiting release of the excitatory neurotransmitter in spinal cord.

Spinal dorsal horn neurons are reactive to nociceptive stimuli and participate in the transmission of painful information to the brain. *Fos* was a proto-oncogene expressed in neurons, and its rapid and transient expression had been identified as an indicator of neuronal excitation (Morgan et al., 1987). In the spinal cord, Fos expression was one of the long-term intracellular events, which was described as an indirect marker of nociceptive processes (Chapman and Besson, 1997). Our result demonstrated CFA injection up-regulated *Fos* mRNA expression in lumbar spinal cord and increased Fos-positive staining of laminae III and IV/V of the dorsal horn compared with the vehicle control, which was consistent with previous studies (Bao et al., 2017; Choi et al., 2018). In this study, we found that i.t. apelin-13 reduced the increased *Fos* mRNA level in lumbar spinal cord and the number of FLI cells located in laminae III and IV/V, suggesting that the inhibitory effect of apelin-13 on inﬂammatory pain was related to the reduction of neuronal activity in spinal dorsal horn.

## Conclusion

We found that i.t. apelin-13 (1, 10 nmol/mouse) alleviated hyperalgesia in response to thermal and mechanical stimulation in a model of chronic inﬂammatory pain induced by injecting CFA into one hind paw of each mouse. Apelin-13 exhibited more potent antinociceptive activity than apelin-36 and (pyr)apelin-13. The antinociception was blocked by APJ antagonist apelin-13(F13A). Additionally, i.t. apelin-13 restored the decreased *Aplnr* mRNA, and reduced the up-regulated Glu concentration, and NR2B and Fos gene and protein level induced by CFA. We suppose that the antinociception of apelin-13 potentially was mediated by APJ activity, and inhibiting the Glu/NR2B function and neural activity of the spinal dorsal horn. Apelin-13 is potentially a preclinical drug for the treatment of inflammatory pain.

## Data Availability Statement

The datasets generated for this study are available on request to the corresponding authors.

## Ethics Statement

The animal study was reviewed and approved by Committee of Medical Ethics and Welfare for Experimental Animals, Henan University School of Medicine (Approval No. HUSOM2016-042).

## Author Contributions

YY and XW developed the idea and designed the research. SL, XZ, YZ, and YF performed the experiment and analyzed the data. SL wrote the draft of the manuscript. YY and XW contributed to revise the writing. All authors contributed to the article and approved the submitted version.

## Funding

This work was supported by the National Natural Science Foundation of China (Grant No. 81600974, and No. 81971280), the Key Science and Technology Program of Henan Province in China (Grant No. 192102310080), the Youth Talent Promotion Plan of Henan Association for Science and Technology (Grant No. 2020HYTP054), Research Program for Young Talent of Henan University School of Medicine (Grant No. 2019018) to SL, the Key Science and Technology Program of Henan Province in China (Grant No. 202102310213) to YY, and the National Natural Science Foundation of China (Grant No. 81771307) to XW.

## Conflict of Interest

The authors declare that the research was conducted in the absence of any commercial or financial relationships that could be construed as a potential conflict of interest.

## Abbreviations

CFA, complete Freund’s adjuvant; CNS, central nervous system; Creb1, cAMP-response element binding protein 1; Dr1, down-regulator of transcription 1; Egr1, Early growth response 1; FLI, c-Fos-like immunoreactive; Fos, FBJ osteosarcoma oncogene; Glu, glutamic acid; GPCR, G protein-coupled receptor; HPA, hypothalamic–pituitary–adrenal; HPLC, high performance liquid chromatography; i.c.v., intracerebroventricular; i.t., intrathecal; NR2B, NMDA Receptor 2B; NF-κB, nuclear factor κB; NMDARs, N-methyl-D-aspartate receptors; PWL, paw withdrawal latency; PWT, paw withdrawal threshold; RT-qPCR, real-time quantitative polymerase chain reaction; SDS-PAGE, sodium dodecyl sulfate- poly acrylamide gel electrophoresis; SIRT1, silent mating type information regulation 2 homolog 1.
